# Integrating Epigenetics, Proteomics, and Metabolomics to Reveal the Involvement of Wnt/β-Catenin Signaling Pathway in Oridonin-Induced Reproductive Toxicity

**DOI:** 10.3390/toxics12050339

**Published:** 2024-05-07

**Authors:** Qibin Wu, Xinyue Gao, Yifan Lin, Caijin Wu, Jian Zhang, Mengting Chen, Jiaxin Wen, Yajiao Wu, Kun Tian, Wenqiang Bao, Pengming Sun, An Zhu

**Affiliations:** 1 Laboratory of Gynecologic Oncology, Department of Gynecology, Fujian Maternity and Child Health Hospital, College of Clinical Medicine for Obstetrics & Gynecology and Pediatrics, Fujian Medical University, Fuzhou 350108, China; 2 Fujian Key Laboratory of Women and Children’s Critical Diseases Research, Fujian Clinical Research Center for Gynecological Oncology, Fujian Maternity and Child Health Hospital (Fujian Women and Children’s Hospital), Fuzhou 350108, China; 3 Key Laboratory of Ministry of Education for Gastrointestinal Cancer, School of Basic Medical Sciences, Fujian Medical University, Fuzhou 350108, China; 4 School of Public Health, Fujian Medical University, Fuzhou 350108, China

**Keywords:** oridonin, reproductive toxicity, Wnt/β-catenin signaling pathway, tight junction, mitochondrial dysfunction

## Abstract

Oridonin is the primary active component in the traditional Chinese medicine *Rabdosia rubescens*, displaying anti-inflammatory, anti-tumor, and antibacterial effects. It is widely employed in clinical therapy for acute and chronic pharyngitis, tonsillitis, as well as bronchitis. Nevertheless, the clinical application of oridonin is significantly restricted due to its reproductive toxicity, with the exact mechanism remaining unclear. The aim of this study was to investigate the mechanism of oridonin-induced damage to HTR-8/SVneo cells. Through the integration of epigenetics, proteomics, and metabolomics methodologies, the mechanisms of oridonin-induced reproductive toxicity were discovered and confirmed through fluorescence imaging, RT-qPCR, and Western blotting. Experimental findings indicated that oridonin altered m6A levels, gene and protein expression levels, along with metabolite levels within the cells. Additionally, oridonin triggered oxidative stress and mitochondrial damage, leading to a notable decrease in WNT6, β-catenin, CLDN1, CCND1, and ZO-1 protein levels. This implied that the inhibition of the Wnt/β-catenin signaling pathway and disruption of tight junction might be attributed to the cytotoxicity induced by oridonin and mitochondrial dysfunction, ultimately resulting in damage to HTR-8/SVneo cells.

## 1. Introduction

Oridonin, characterized by a tetracyclic diterpenoid structure with isoprene as its core framework, is recognized as the primary bioactive constituent of the traditional Chinese medicine *Rabdosia rubescens* [1]. Studies have demonstrated the potent pharmacological properties of oridonin, including anti-inflammatory, anti-tumor, and antibacterial effects [2,3]. It is frequently prescribed for treating pharyngitis, tonsillitis, and bronchitis in clinical practice [4]. Conversely, an increasing amount of research has been dedicated to exploring the adverse reactions associated with oridonin. After 21 days of continuous injection of 10 mg/kg of oridonin in healthy nude mice, it led to hepatic sinusoidal constriction and a significant increase in alanine aminotransferase level [5]. Treatment with 10 μM of oridonin for 24 h resulted in DNA damage and cellular accumulation of reactive oxygen species (ROS), promoting H460 human lung epithelial cell apoptosis [6]. Notably, after treating ovarian granulosa cells with 15 μg/mL of oridonin for 24 h, it was observed that cell proliferation was inhibited, SOD activity decreased, and the levels of malondialdehyde and ROS increased, resulting in oxidative damage to the ovarian granulosa cells [7]. However, few studies have been conducted on the reproductive toxicity of oridonin, and its potential toxicity mechanism remains unclear, which hinders its clinical application.

The mitochondria serve as the central hub for cellular energy synthesis and represent the primary source of ROS. Given the intricate structure and diverse functions of mitochondria, numerous mechanisms can contribute to mitochondrial toxicity triggered by drugs and other chemical compounds [8]. Previous research has demonstrated that the combination of AG1478 and oridonin enhances ROS generation. Cells treated with n-acetylcysteine during incubation mitigated apoptosis and prevented disruption of mitochondrial membrane potential (MMP) caused by the combined treatment of AG1478 and oridonin, implying that ROS are crucial in mediating cell death triggered by oridonin [9]. Furthermore, given the limited protective system against oxidative stress, mitochondria are anticipated to be prone to oxidative damage [10]. It has been firmly established that an excess of ROS is responsible for impeding the respiratory chain complexes, particularly complex I, as well as ATP synthesis. Additionally, ROS causes the oxidation of lipid peroxidation, and protein oxidation. These consequences ultimately trigger the initiation of the mitochondrial permeability transition pore (mPTP) and a subsequent reduction in MMP [11,12]. During embryogenesis, WNTs act as signaling molecules that regulate essential processes such as cell division, tissue morphogenesis, and cell development. By binding to specific receptors on neighboring cells, WNTs activate intracellular signaling pathways, leading to changes in gene expression and cellular homeostasis [13]. The Wnt signaling pathway primarily refers to mediation by the activation of target genes and the nuclear translocation of β-catenin [14]. Regulation of the cytoskeleton, adhesion, and migration involves the Wnt signaling via the Wnt/Ca^2+^ and Wnt/PCP pathways [15]. By modulating the β-catenin stability, a crucial constituent of the adherens junction, canonical Wnt signaling significantly impacts the regulation of cell adhesion [16]. The upregulation of Wnt-5a led to enhanced cell–substrate adhesion, cell migration, and focal adhesion kinase activation dependent on adhesion in cancer cells [17]. The tumor suppressor protein APC plays a crucial role in the Wnt signaling pathway by coordinating cytoskeletal networks. By binding to the plasma membrane in an actin-dependent fashion, APC modulates the actin cytoskeleton, which is necessary for cell polarization and directed migration [18]. Overexpression of CLDN1, which serves as a primary element within tight junction complexes governing the permeability of epithelial barriers in the colorectal cancer mouse model, led to decreased survival. Transcriptome analysis supported an upregulation in the Wnt signaling pathway [19]. However, the exact mechanism behind oridonin-induced reproductive toxicity is not thoroughly elucidated and there is no evidence of a relationship between its toxicity to mitochondria and Wnt/β-catenin signaling pathway.

The advancement of omics methodologies in recent years has been crucial in driving progress in the field of biology, with the ultimate goal of unraveling intricate mechanisms underlying complex phenomena. Instead of employing conventional biochemical methods to analyze individual components of an organism, the omics approach encompasses a comprehensive examination of all components and their intricate interconnections throughout the entire system. Omics technology is commonly used in traditional Chinese medicine and other ethnomedical practices. Genomics, transcriptomics, proteomics, and metabolomics are increasingly integral to pharmaceutical research, often used individually or in combination. The application of omics technologies is becoming more prevalent, with its effectiveness acknowledged in target discovery, toxicology studies, and traditional Chinese medicine research [20]. Epigenetics primarily encompasses the investigation of heritable variations in cell phenotype or gene expression that occur without alterations to the nucleotide sequence. Based on the extent of modification, it can be divided into transcriptional, post-transcriptional, and post-translational modifications [21,22]. RNA modifications also exert regulatory functions at the post-transcriptional level. More than 170 RNA modifications have been discovered [23], with N6-methyladenosine (m6A) being widely acknowledged as the most prevalent and abundant modification [24,25]. With the advancement of our comprehension regarding regulatory mechanisms, it has become evident that m6A methylation is critical in various stages of RNA metabolism [26]. Recent research has emphasized the crucial role of m6A methylation modifications in orchestrating diverse cellular processes, encompassing the regulation of cell proliferation and differentiation [27], DNA damage response regulation [28], and cellular autophagy modulation [29]. For instance, the collaborative involvement of methyltransferase 3, N6-adenosine-methyltransferase complex catalytic subunit (METTL3), and fat mass and obesity (FTO)-mediated m6A modifications is observed in the cadmium sulfate-induced oxidative stress process, leading to cellular apoptosis [30]. The downregulation of METTL3 modulates the response to oxidative stress in mouse renal tubular epithelial cell models when exposed to colistin [31]. In addition, advancements in proteomics have significantly broadened its applications in the field of biomedicine, enabling specific identification and quantification of proteins within organisms [32]. The utilization of proteomics shows great potential in the realm of drug development, particularly in identifying drug targets, exploring drug mechanisms, and conducting toxicology research. Gambogic acid and gambogenic acid, two derivatives of Gamboge, have been discovered to be capable of inhibiting the target protein stathmin 1 in HepG2 cell proliferation [33]. Metabolomics, an emerging omics technology that follows transcriptomics and proteomics, investigates endogenous metabolites of small molecules within organisms to explore organismal metabolic states and enhance the comprehensive understanding of the entire system when integrated with other omics approaches [34]. Metabolites serve as the ultimate products of biological processes, enabling metabolomic findings to depict prior toxicological events. In a metabolomic investigation, nephrotoxicity induced by aristolochic acid was characterized by the significant acceleration of specific metabolic pathways such as the folate cycle and homocysteine synthesis, alongside a decrease in activity in pathways such as arachidonic acid biosynthesis [35]. By integrating assessments of the epigenetic transcriptome, proteome, and metabolome, potential biomarkers within crucial signaling pathways can be effectively discerned.

In the present study, the reproductive toxicity of oridonin was evaluated on HTR-8/SVneo cells and potential molecular mechanisms were demonstrated. It was found that there is a dose-dependent inhibition of viability in HTR-8/SVneo cells by oridonin. Moreover, oridonin demonstrated damage to mitochondria and disruption of tight junction. Mechanistically, our study suggested that oridonin might mediate its reproductive toxicity by suppressing the activation of the Wnt/β-catenin signaling pathway.

## 2. Materials and Methods

### 2.1. Chemical Reagent and Cell Culture

**Figure 1:**
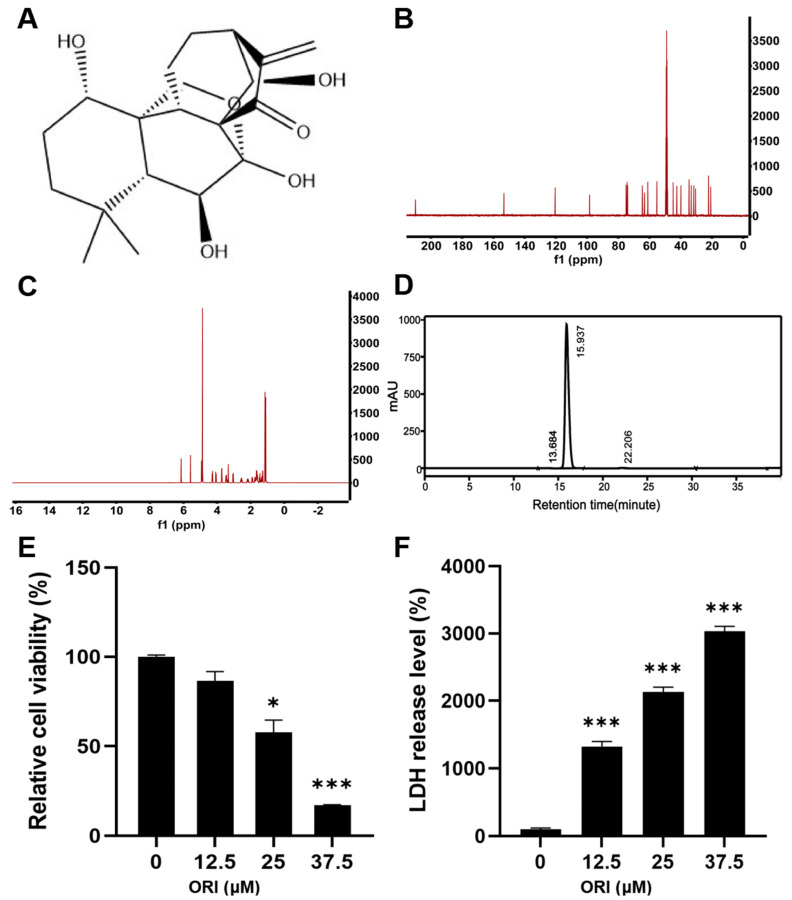
(**A**) The chemical structure of oridonin. (**B**) 400 MHz ^13^C NMR spectra of oridonin. (**C**) 400 MHz ^1^H NMR spectra of oridonin. (**D**) Purity analysis of oridonin by HPLC. The viability (**E**) and LDH release level (**F**) of HTR-8/SVneo cells following treatment with 0, 12.5, 25, and 37.5 μM oridonin for 24 h. * *p* < 0.05, *** *p* < 0.001, n = 3.

Oridonin, provided by Chengdu Must Bio-Technology Co., Ltd. (Chengdu, China), boasted a purity of 99.64% (Figure 1D). The purity and chemical structure (Figure 1A) of oridonin were verified through high-performance liquid chromatography (HPLC) and 400 MHz ^13^C (Figure 1B) and ^1^H (Figure 1C) nuclear magnetic resonance spectroscopy, respectively. The human trophoblast cell line HTR-8/SVneo was sourced from the American Type Culture Collection and cultured in DMEM supplemented with 100 μg/mL streptomycin, 100 U/mL penicillin G sodium salt, and 10% fetal bovine serum. The maintenance conditions included a temperature of 37 °C and 5% CO_2_. To treat HTR-8/SVneo cells, it was dissolved in DMSO at concentrations of 12.5, 25, and 37.5 μM in well plates for 24 h. The concentration of DMSO in the cell culture was maintained at 1%.

### 2.2. MTT and LDH Release Assay for Determining Cytotoxic Effects

The evaluation of HTR-8/SVneo cell viability following exposure to oridonin was carried out through the MTT cytotoxicity assay. Briefly, 4 × 10^3^ cells were plated in individual wells of 96-well plates and exposed to 0, 12.5, 25, and 37.5 μM oridonin for 24 h. Subsequently, each well received 10% MTT solution and was then incubated in a 37 °C cell culture incubator for 4 h. Post-medium removal, 100 μL DMSO was added to each well to dissolve the intracellular formazan product. The absorbance of formazan was determined at 490 nm with a microplate reader (BioTek, Santa Clara, CA, USA).

The assessment of cell membrane integrity included quantifying the release of LDH. Following a 24 h treatment with 0, 12.5, 25, and 37.5 μM oridonin, the 96-well plate was centrifuged at 400× *g* for 5 min. Subsequently, a volume of 120 μL supernatant was transferred to a new 96-well culture plate and incubated with LDH detection reagent for 30 min. The release level of LDH was assessed using a microplate reader (BioTek) at a wavelength of 490 nm.

### 2.3. RNA Extraction and High-Throughput Sequence

The HTR-8/SVneo cells were exposed to 25 μM oridonin for 24 h, followed by washing with PBS. Subsequently, total RNA was isolated using Trizol reagent (Invitrogen, Carlsbad, CA, USA), followed by DNaseI treatment to remove DNA. The quality of samples was evaluated by a Nanodrop One C spectrophotometer (Thermo Fisher, Waltham, MA, USA), while the integrity was assessed using agarose gel electrophoresis. A total of 50 μg eligible RNA was used for high-throughput sequence, including m6A MeRIP-seq and mRNA-seq, conducted by Seqhealth (Wuhan, China). To address the issue of duplication bias during RNA library establishment, the KC-Digital Stranded mRNA Library Prep Kit for Illumina (Seqhealth) was applied. The resulting library fragments, ranging from 200 to 500 bps, were enriched and quantified. The comparison between the oridonin group and the control group was conducted using fold change and *p*-value. To obtain RIP-Seq peaks, motifs, and data analysis of Kyoto Encyclopedia of Genes and Genomes (KEGG) and Gene Ontology (GO), the data were inputted into exomePeak (Suzhou, China), Homer (San Diego, CA, USA), and DAVID (Frederick, MD, USA). Each group was sequenced with three independent samples.

Quality control of mRNA-seq data is shown in Table S1. The control groups obtained average quantities of 75.1 million raw reads of mRNA, with 68.1 million clean reads. Meanwhile, the oridonin groups obtained average quantities of 71.0 million raw reads of mRNA, with 64.1 million clean reads. The Q20 was 99.4% and Q30 was 97.0% in all samples. The above results suggest that our mRNA-seq was of high quality.

### 2.4. Molecular Docking and Molecular Dynamics (MD) Simulation

The interaction between oridonin and m6A regulators was explored through SYBYL software (Tripos, St Louis, MO, USA). The structure of oridonin was obtained from PubChem, subjected to energy minimization, and converted into 3D structures using Chem3D (Waltham, MA, USA) [36]. The crystalline structure of docking proteins was acquired from PDB. Then, the high-precision and semi-flexible docking was performed in SYBYL-X 2.0. A stable binding between proteins and molecules was considered if the total score surpassed 5 [37].

To investigate protein–ligand interactions, we employed the Simulation Package tOward Next GEneration (SPONGE, Beijing, China) [38]. Throughout the simulation study, the FF14SB united-atom force field was utilized [39]. The resolution of complex systems involved the utilization of the SPC/E water model within cubic boxes [40], keeping a minimum distance of 1.2 nm from the box edges. Neutralization was achieved by introducing potassium and chloride ions, while periodic boundary conditions were imposed. Energy minimizations were computed utilizing the steepest descent algorithm with a tolerance of 5.0 kJ/mol. Subsequently, a 100 ps NVT equilibration was executed at 300 K employing Langevin thermostat temperature coupling, with relaxation time constants set at 1.0 ps. This was followed by a 100 ps NPT simulation at 1 bar, performed utilizing Andersen barostat in conjunction with Nosé–Hoover thermostat to maintain constant pressure. Subsequently, 50 ns MD simulations were conducted for each system. Various MD trajectory analyses were employed to evaluate binding stability under dynamic conditions.

### 2.5. Proteomics Sample Processing and LC-MS/MS Analysis

After exposure to 25 µM oridonin for 24 h, cells were lysed using a lysis buffer comprising 100 mM triethyl-ammonium bicarbonate and 1% SDS. After centrifugation at 12,000× *g* for 10 min, the supernatant was subjected to protein concentration measurement using bicinchoninic acid (BCA) assays. A total of 150 μg protein underwent reduction and alkylation with 200 mM Tris (2-carboxyethyl) phosphine hydrochloride and 375 mM iodoacetamide, respectively. After pre-cooled acetone treatment, the precipitate was accumulated through a 5 min centrifugation and then dried in air. Following mixing with a 200 mM triethylammonium bicarbonate buffer, the samples were incubated with trypsin overnight at 37 °C.

After labeling, blending, desalting, and vacuum-drying, the peptides were partitioned into 15 fractions employing the high-pH reversed-phase peptide fractionation kit (Thermo Fisher). The 0.1% formic acid was used for dissolution processing, loaded directly onto a C18, 75 μm × 25 cm reversed-phase analytical column (Thermo Fisher) for LC-MS/MS analysis. The output data underwent processing in Proteome Discoverer version 3.0, where it was matched against the mus musculus Uniprot database. Methionine oxidation and protein N-terminus acetylation were considered as the variable modification, while fixed modifications were specified as carbamidomethyl for cysteine and TMTpro for lysine and the N-terminus.

### 2.6. Sample Preparation and Analysis for Metabolomics

HTR-8/SVneo cells, exposed to 25 µM oridonin for 24 h, were washed with PBS and flash-frozen in liquid nitrogen before being prepared for UPLC-MS/MS analysis performed by Metware (Wuhan, China). The cells were then treated with 300 μL methanol extract containing 20% acetonitrile and vortexed. After centrifugation at 12,000 rpm for 10 min to separate the supernatant, the samples were incubated at −20 °C for 30 min. A further centrifugation under the same conditions was performed to collect supernatant.

Metabolites were separated using a 1.8 µm, 2.1 mm × 100 mm ACQUITY UPLC HSS T3 C18 column (Waters, Milford, MA, USA), under the conditions of an injection volume of 2 μL, flow rate of 0.4 mL/min, and a column temperature maintained at 40 °C. The solvent setup comprised water containing 0.1% formic acid (mobile phase A) and acetonitrile with 0.1% formic acid (mobile phase B). The linear gradient began at 5% to 90% mobile phase B over an 11 min period, followed by holding it for an additional minute before returning to the initial composition within just 0.1 min and maintaining it for another 1.9 min. After separation, the nanoparticles were ionized and analyzed using a Triple TOF-6600 mass spectrometer (AB Sciex, Foster City, CA, USA). The resulting data were cross-referenced with the NIST Chemistry WebBook, the Human Metabolome Database, as well as various personal and public databases. Statistical algorithms were then employed to process the data for missing value supplementation and fold change calculation of metabolites. Subsequently, significantly different metabolites were identified for further multi-level bioinformatics and functional analysis.

### 2.7. ROS Measurement

Cellular ROS levels were quantified utilizing an ROS assay kit (Solarbio, Beijing, China) following the provided guideline. In brief, cells were treated with oridonin at concentrations of 0, 12.5, 25, and 37.5 μM and subsequently exposed to DCFH-DA (10 μM) at 37 °C for 30 min, followed by rinsing with PBS. Afterwards, visualization of intracellular fluorescence was accomplished utilizing a fluorescence microscope (Zeiss, Oberko-chen, Germany), and quantification of fluorescence intensity was analyzed using ImageJ software (Bio-Rad, Hercules, CA, USA).

### 2.8. DNA Damage Assay by γ-H2AX Immunofluorescence

The DNA damage assay kit (Beyotime, Shanghai, China) was utilized to assess the immunofluorescence of γ-H2AX. HTR-8/SVneo cells were exposed to oridonin at concentrations of 0, 12.5, 25, and 37.5 μM for 24 h. Cells were fixed and blocked at room temperature for 15 min. The cells underwent incubation with a rabbit anti-γ-H2AX primary antibody and corresponding secondary antibody for 1 h. Subsequently, nuclear staining was conducted using 4′,6-diamidino-2-phenylindole (DAPI). Ultimately, the green fluorescence images were captured by a microscope (Zeiss) at 519 nm.

### 2.9. Detection of Calcium by Fluo-4 AM

Intracellular Ca^2+^ levels in HTR-8/SVneo cells were detected using Fluo-4 AM (Beyotime), a fluorescence probe that can penetrate cell membranes. Cells were cultured with 0, 12.5, 25, and 37.5 μM oridonin for 24 h and washed thrice in PBS. Fluo-4 AM was diluted to 1 μM working solution with PBS and used to incubate cells at 37 °C for 1 h. Following a single wash, the fluorescence of Ca^2+^ was observed using a fluorescence microscope (Zeiss).

### 2.10. Detection of MMP and mPTP

The MMP was assessed using the JC-10 probe (Solarbio). In general, the MMP is in a relatively high state. Within the mitochondria matrix, the JC-10 probe forms polymers that result in red fluorescence. When the mitochondria are damaged, the membrane potential decreases. At this point, JC-10 cannot aggregate in the matrix and exists as monomers, producing green fluorescence. This shift from red to green fluorescence with the JC-10 probe enables easy detection of MMP reduction. The HTR-8/SVneo cells were exposed to concentrations of 0, 12.5, 25, and 37.5 μM oridonin for 24 h. Subsequently, the cells were incubated with the JC-10 probe under dark conditions at 37 °C for 20 min. Finally, red and green fluorescence were analyzed using a microscope (Zeiss).

The mPTP opening of HTR-8/SVneo cells in each group was evaluated using Calcein AM probe (Beyotime). The mPTP is a non-selective channel formed by both the inner and outer mitochondrial membranes and plays a role in substance release from the mitochondria during cell death. The Calcein AM probe can passively enter the cell and accumulate in cellular components, including the cytoplasm and mitochondria. Within the cell, the nearly non-fluorescent Calcein AM undergoes hydrolysis by intracellular esterases to remove the acetyl methyl ester, leading to the generation of the membrane-impermeable polar fluorescent dye calcein. This allows Calcein to be retained within the cell, emitting intense green fluorescence. Calcein is also utilized as a metal chelating agent. When the opening degree of the mPTP increases, it complexes with metal ions such as Co^2+^, causing fluorescence signal quenching. Therefore, CoCl_2_ serves as a positive control for mPTP detection. The HTR-8/SVneo cells were exposed to oridonin (0, 12.5, 25, and 37.5 μM) for 24 h. Afterward, they were incubated with the Calcein AM probe at 37 °C in the dark for 40 min. The staining solution was then substituted with warm culture medium, and the cells were cultured for an additional 30 min. Subsequently, Hoechst 33342 was used to stain the nuclei for 10 min, and the fluorescence was observed under a fluorescence microscope (Zeiss).

### 2.11. RT-qPCR

The HTR-8/Svneo cells were exposed to concentrations of 0, 12.5, 25, and 37.5 μM oridonin for 24 h. As previously specified, the RNA extraction was subsequently executed, quantified to 1 μg. Then, the RNA underwent reverse transcription into cDNA using Evo M-MLV reverse transcription premixed kit (Accurate Biology, Changsha, China) for RT-qPCR. The reaction took place in a 20 μL volume, with 10 μL SYBR Green qPCR Mix, 2 μL template, and 10 μM primers (Monad, Suzhou, China). The relative gene expression under oridonin treatment was determined utilizing the 2^−ΔΔCt^ method [41], with the fold change compared to the control group. Table 1 displays the RT-qPCR primer sequences employed in this investigation. *GAPDH* was designated as the endogenous control for the remaining target genes.

### 2.12. Western Blotting Assay

Cells exposed to oridonin with 0, 12.5, 25, and 37.5 μM for 24 h were collected and lysed using RIPA buffer (Beyotime) containing a phosphatase inhibitor cocktail (Beyotime) and PMSF for protein extraction. The BCA protein assay kit (Vazyme, Nanjing, China) was used to quantify total protein concentration. Protein samples were separated through electrophoresis on 10% sodium dodecyl sulphate–polyacrylamide gel and subsequently transferred onto polyvinylidene fluoride (PVDF) membranes (Millipore, Billerica, MA, USA) for 90 min. The membranes were then blocked by 5% skimmed milk at room temperature for 90 min and then incubated with primary antibodies, including WNT6 (Proteintech, Wuhan, China), β-catenin (Proteintech), GSK3B (Proteintech), CCND1 (Proteintech), CLDN1 (Proteintech), ZO-1 (Proteintech), and TCF7L1 (Proteintech), which were used at a dilution of 1:1000, 1:10,000, 1:4500, 1:10,000, 1:4500, 1:10,000, and 1:1000, respectively. GAPDH (ABclonal, Wuhan, China) was used as internal control at a dilution of 1:10,000. Following primary antibody incubation, PVDF membranes were incubated with either goat anti-mouse IgG secondary antibody or horseradish peroxidase-conjugated goat anti-rabbit IgG secondary antibody (both at a dilution of 1:10,000; Proteintech) at room temperature for 90 min. Visualization of protein bands was carried out using the Amersham Imager 680 (GE Healthcare Bio-Sciences AB, Uppsala, Sweden). The Image J software (Bio-Rad) was utilized for quantitative analysis of the target bands.

### 2.13. Statistical Analysis

SPSS 26 (IBM, New York, NY, USA) was used for conducting the data analysis. The test of homogeneity of variances and one-way analysis of variance (ANOVA) were employed to assess variance consistency and group differences, respectively. Data were presented as mean ± standard error of the mean (SEM). Statistically significant ANOVA results were determined by *p*-values < 0.05.

## 3. Results

### 3.1. The Cytotoxic Effect of Oridonin on HTR-8/SVneo Cells

Cell viability of HTR-8/SVneo cells was examined through the MTT assay. A concentration-dependent inhibition of cell viability was observed for oridonin, as illustrated in Figure 1E. Exposure to 37.5 μM oridonin for 24 h resulted in a cell viability of 17% for HTR-8/SVneo cells. Moreover, at concentrations of 12.5 and 25 μM, cell viability decreased to 86.4% and 57.7%, respectively.

LDH level serves as a crucial indicator for assessing the integrity of cell membranes. Quantitative analysis of cytotoxicity can be carried out by measuring the amount of LDH released into the supernatant due to membrane rupture. As depicted in Figure 1F, treatment with oridonin resulted in an elevation in the LDH release, suggesting that oridonin compromised cell integrity and caused cellular damage.

### 3.2. Transcriptome-Wide Assessment of m6A Modification Post-Oridonin Treatment in HTR-8/SVneo Cells

**Figure 2:**
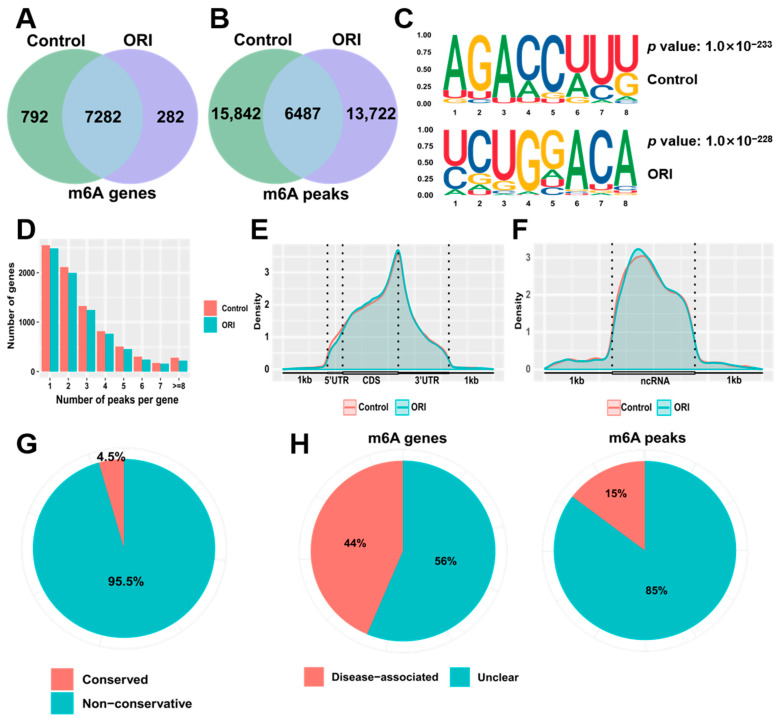
Illustration in a Venn diagram depicting the overlap of m6A-modified genes (**A**) and peaks (**B**) between oridonin and control groups. (**C**) Analysis of m6A motifs in the oridonin and control groups. (**D**) Distribution pattern of m6A modification peaks per gene. Comparing the difference in density of m6A-modified peaks in mRNA (**E**) and ncRNA (**F**). Identification of conserved m6A sites (**G**), m6A genes and peaks related to the disease (**H**) in differentially modified m6A-mRNAs.

RNA epigenetic modifications, particularly m6A, have been reported to be involved in gene expression regulation. We performed MeRIP-seq on both oridonin-treated cells and control cells that were untreated to analyze the changes in m6A modification following oridonin treatment. The analysis revealed 22,329 peaks across 8074 genes in the control group, while 20,209 m6A peaks were identified in 7564 m6A genes in the oridonin group (Figure 2A,B). STREME analysis revealed that the mammalian m6A conserved modification motif RRACH was enriched in both the control and oridonin groups, with R representing adenine or guanine and H representing adenine, uracil, or cytosine (Figure 2C). Transcripts containing m6A modifications were classified based on the quantity of modification peaks observed in each transcript. The analysis revealed that over 3000 genes displayed 1–2 m6A modification peaks in every group, while a relatively limited number of transcripts showed an increased occurrence of more than 4 m6A modification peaks (Figure 2D). m6A modification on transcripts was not randomly distributed. Figure 2E shows that m6A modifications were primarily concentrated in the coding region sequence and the 3′ untranslated region of mRNA, and were highly enriched in the stop codon region. Interestingly, m6A modifications were also abundant in non-coding RNA (Figure 2F).

By conducting ConsRM and RMDisease analyses, we explored the conservation of m6A and its association with diseases. The ConsRM analysis showed that 95.5% of m6A-modified sites exhibited non-conservative characteristics in differentially modified m6A and differentially expressed genes (DEGs) (Figure 2G). Additionally, the RMDisease analysis indicated that 15% of m6A peaks and 44% of m6A genes were linked to various diseases (Figure 2H).

### 3.3. Combined Analysis of RNA-seq and MeRIP-seq

**Figure 3:**
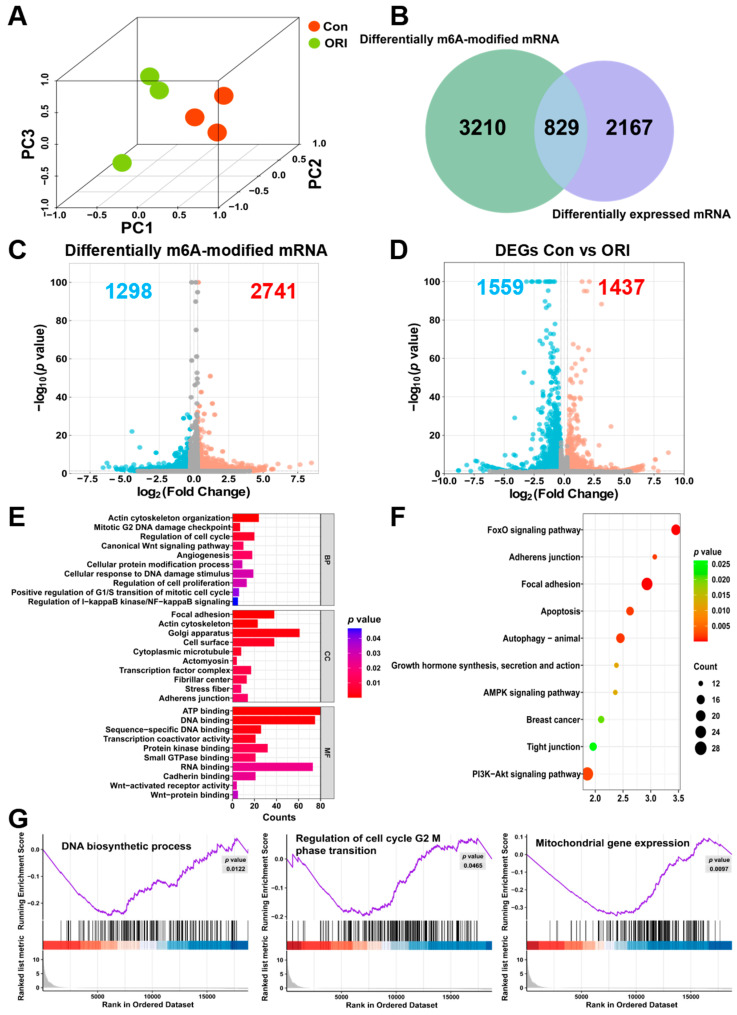
Molecular diversity of HTR-8/SVneo cells post-treatment with oridonin. (**A**) PCA for the mRNA expression between the oridonin and control groups. (**B**) Venn plot illustrating the intersection between differentially modified m6A genes and DEGs. (**C**) Volcano plot depicting genes m6A methylation downregulated (in blue) or upregulated (in red). (**D**) Volcano plot depicting genes expression downregulated (in blue) or upregulated (in red). The GO (**E**) and KEGG (**F**) enrichment of 829 overlapped genes among DEGs and differentially m6A-methylated mRNAs. (**G**) GSEA revealed that genes in oridonin group were enriched for DNA biosynthetic process, regulation of cell cycle G2/M phase transition, and mitochondrial gene expression.

To demonstrate the regulatory effect of m6A modification on the Wnt signaling pathway, this study combined RNA-seq and MeRIP-seq data analysis to identify genes with notable changes at both mRNA expression and m6A modification. The PCA results demonstrated marked distinctions among samples from the oridonin and control groups, with well-clustered samples within each group indicating that gene expression levels were significantly altered after oridonin treatment of HTR-8/SVneo cells, and also confirmed the reliability of the sequencing data (Figure 3A). The screening criteria for DEGs and differentially modified m6A genes were set as fold change > 1.2 or <0.83, with *p* < 0.05. A total of 2296 DEGs were detected by mRNA-seq, with 1437 genes upregulated and 1559 genes downregulated. MeRIP-seq identified a total of 4039 differentially modified m6A genes, including 2741 genes with enhanced m6A modification and 1298 genes with weakened m6A modification (Figure 3C,D). Notably, 829 genes showed significant changes at both levels (Figure 3B). The 4039 differentially modified m6A genes, along with their *p*-values and fold changes, as well as the 2296 DEGs with their corresponding *p*-values and fold changes, are listed in Table S2 and Table S3, respectively.

Subsequently, 829 overlapping genes were analyzed for KEGG and GO enrichment. GO enrichment analysis revealed that these genes were mainly enriched in regulation of cell cycle, cellular response to DNA damage stimulus, regulation of cell proliferation, adherens junction, focal adhesion, Wnt-activated receptor activity, and Wnt-protein binding (Figure 3E). KEGG enrichment results mainly included focal adhesion, adherens junction, growth hormone synthesis, secretion and action, and tight junction (Figure 3F). We conducted gene set enrichment analysis (GSEA) to further clarify the signaling pathways linked to oridonin treatment. Comparative GSEA analysis between the oridonin and control groups indicated that regulation of cell cycle G2/M phase transition, DNA biosynthetic process, and mitochondrial gene expression were inhibited (Figure 3G).

### 3.4. Potential Regulators of RNA m6A Methylation

An analysis of mRNA expression levels for 22 RNA m6A methylation regulators was conducted to identify potential regulators using mRNA-seq data. As shown in Table 2, writers *RBM15*, *RBM15B*, *WTAP*, and reader *IGF2BP2* were significantly downregulated, while readers *FMR1*, *HNRNPA2B1*, and *YTHDF2* were significantly upregulated.

**Figure 4:**
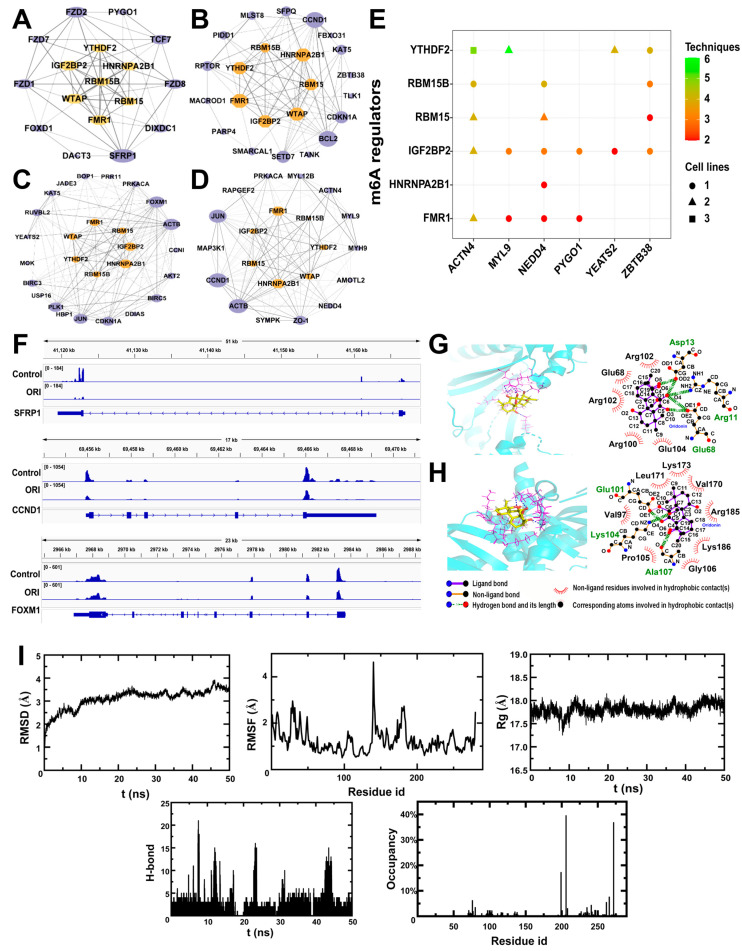
The associations between m6A regulators and the genes of Wnt signaling pathway (**A**), cellular response to DNA damage (**B**), regulation of cell cycle (**C**), and tight junction (**D**). (**E**) The impact of m6A regulators on the DEGs within Wnt signaling pathway, regulation of cell cycle, cellular response to DNA damage, and tight junction. (**F**) The level of m6A modification on *SFRP1*, *CCND1*, and *FOXM1* mRNA transcripts visualized by IGV. The interactions of oridonin with m6A regulators of FMR1 protein (**G**) and HNRNPA2B1 protein (**H**). (**I**) Molecular dynamics simulation between FMR1 protein and oridonin.

We collected genes related to the Wnt signaling pathway, regulation of cell cycle, cellular response to DNA damage, and tight junction from GO and KEGG enrichment results. Subsequently, we utilized STRING to investigate the regulatory connections between these genes and m6A regulators, thereby conducting network analysis for protein–protein interactions. In Figure 4A–D, the top five genes in Wnt signaling pathway term were *SFRP1*, *FZD2*, *TCF7*, *FZD8*, and *FZD1*, with the degrees of 10, 8, 7, 7, and 7 (Figure 4A); the top five genes in cellular response to DNA damage term were *CCND1*, *BCL2*, *CDKN1A*, *SETD7*, and *SFPQ*, with the degrees of 15, 15, 10, 10, and 7 (Figure 4B); in regulation of cell cycle term, the top-ranking genes included *FOXM1*, *ACTB*, *JUN*, *PLK1*, and *CDKN1A*, with the degrees of 20, 20, 17,16, and 15 (Figure 4C); the top five genes in tight junction term were *CCND1*, *ACTB*, *JUN*, *TJP1*, and *ACTN4*, with the degrees of 18, 18, 17, 13, and 10 (Figure 4D).

To explore substrates targeted by RNA m6A methylation regulators, the writers RBM15, RBM15B, WTAP, and readers IGF2BP2, FMR1, HNRNPA2B1, and YTHDF2 were selected, along with genes related to the Wnt signaling pathway, regulation of the cell cycle, cellular response to DNA damage, and tight junction. The results indicated that IGF2BP2 and FMR1 were extensively involved in regulating the majority of genes within these four terms (Figure 4E). Utilizing IGV, the m6A methylation patterns of transcripts were visualized. After treatment with oridonin, the m6A methylation levels of the Wnt signaling pathway-related gene *SFRP1*, regulation of cell cycle-related gene *FOXM1*, and cellular response to DNA damage-related gene *CCND1* decreased (Figure 4F).

### 3.5. Molecular Interactions and Molecular Dynamics between Oridonin and m6A Regulatory Proteins

Predictions of the interactions between oridonin and m6A regulatory proteins are presented in Table 3, Figure 4G,H. Oridonin formed hydrogen bonds with Arg11, Asp13, Arg102, and Glu68 in FMR1 protein, as well as with Glu101, Lys104, and Ala107 in HNRNPA2B1 protein. In Arg100 and Glu104 sites, FMR1 made hydrophobic contacts with oridonin, while in Pro105, Gly106, Lys186, Arg185, Val170, Lys173, Leu171, and Val197 sites, HNRNPA2B1 made hydrophobic contacts with oridonin. The total scores of the above two proteins exceeded 5, suggesting that the proteins were the most likely to bind to oridonin.

In evaluating the binding stability and conformational variability of the FMR1 protein and oridonin complex throughout MD simulation, RMSD, RMSF, Rg, H-bound, and occupancy analyses were performed (Figure 4I). The complex demonstrated average RMSD values spanning from 0.6 to 3.9 Å, with Rg values showing fluctuations within a 1 Å range. The residue-wise fluctuations of FMR1 protein and oridonin were plotted. Analysis of RMSF indicated that no amino acid residues exhibited conformational fluctuations exceeding 4.7 Å relative to their mean structure, highlighting the significant stability of backbone atoms within this complex system. The quantity of hydrogen bonds established throughout the simulation was recorded. Noteworthy is the observation that among the 280 amino acid residues, aspartic acid at position 206 exhibited the highest hydrogen bond occupancy at 40%.

### 3.6. Results of the Analysis of Differentially Expressed Proteins (DEPs)

**Figure 5:**
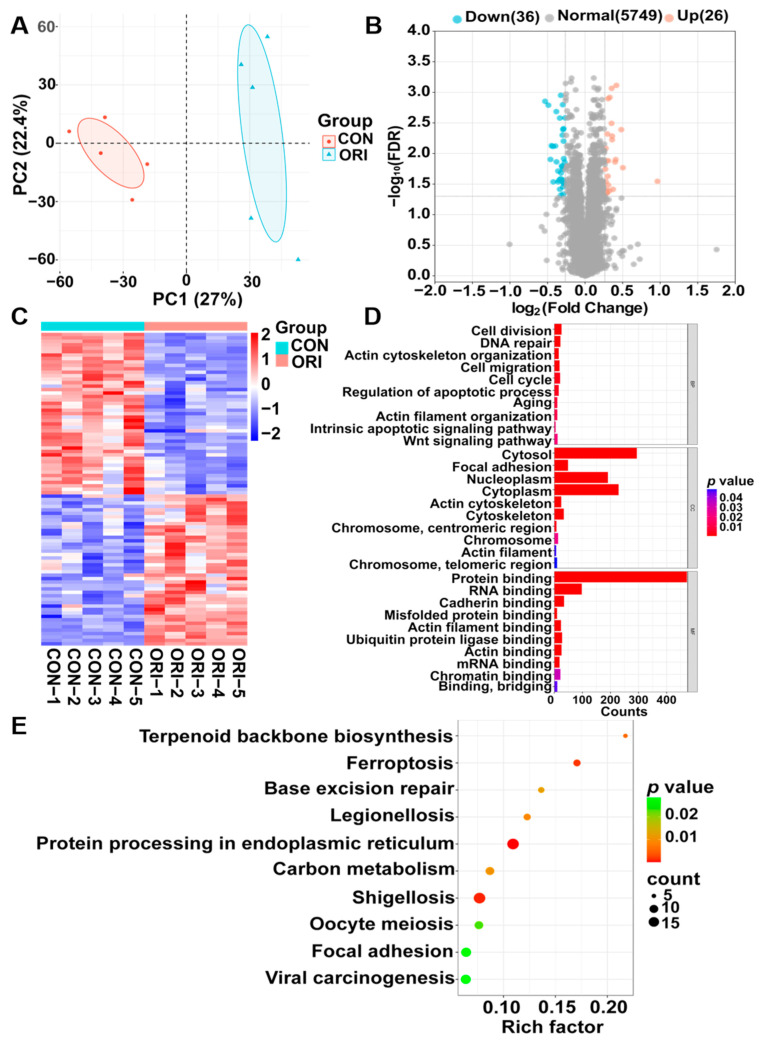
(**A**) PCA for the protein expression in the oridonin and control groups. (**B**) The volcano plot showed the DEPs in the oridonin and control groups. (**C**) Heatmap of DEPs in the oridonin and control groups. Enrichment analysis for GO terms (**D**) and KEGG pathway (**E**) related to DEPs.

The PCA plot between the oridonin and control groups (Figure 5A) shows a significant difference. The volcano plot illustrates the distribution of all proteins identified through the LC-MS/MS approach. Proteins that were significantly downregulated and upregulated (FDR < 0.05, fold change > 1.2 or <0.83) are highlighted in blue and red, while proteins showing non-significant distinctions between the oridonin and control groups are depicted in grey. A total of 62 differentially expressed proteins (DEPs) were screened, with 26 downregulated and 36 upregulated (Figure 5B). The clustering analysis of DEPs is presented in the heatmap (Figure 5C). The 62 DEPs, along with their corresponding FDR and fold changes, are listed in Table S4.

### 3.7. Analysis Results from GO Annotation and Enrichment of KEGG Pathways

Supplementary analyses using GO and KEGG pathways were carried out to examine potential significant enrichment trends of DEPs (FDR < 0.05) in distinct functional categories. The DEPs associated with biological processes (BPs) were mainly enriched in functions associated with cell division, cytoskeleton organization, cell cycle regulation, the Wnt signaling pathway, and apoptosis. In terms of cellular components (CC), the DEPs were significantly enriched in terms such as focal adhesion, actin cytoskeleton, and chromosome. For molecular functions (MFs), terms showed predominant enrichment in actin binding, RNA binding, and cadherin binding, as illustrated in Figure 5D. The KEGG pathway is associated with the terpenoid backbone biosynthesis, ferroptosis, and focal adhesion (Figure 5E).

**Figure 6:**
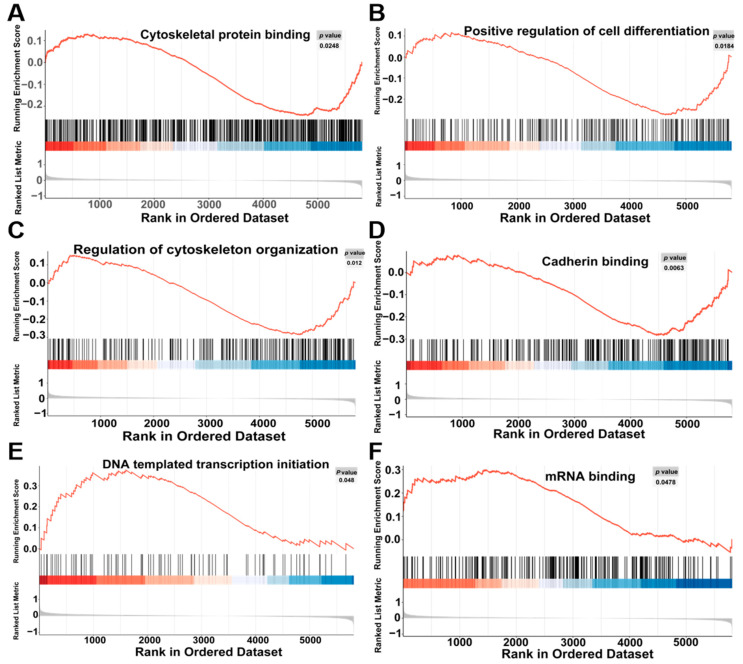
GSEA results between the oridonin and control groups. Cytoskeletal protein binding (**A**), positive regulation of cell differentiation (**B**), regulation of cytoskeleton organization (**C**), cadherin binding (**D**), DNA templated transcription initiation (**E**), and mRNA binding (**F**).

In comparison to the control group, GSEA of oridonin-treated samples revealed inhibition in cytoskeletal protein binding, positive regulation of cell differentiation, regulation of cytoskeleton organization, and cadherin binding, while the DNA templated transcription initiation and mRNA binding were activated (Figure 6A–F).

### 3.8. Analysis of Metabolomics Data between Oridonin-Treated and Control Groups

**Figure 7:**
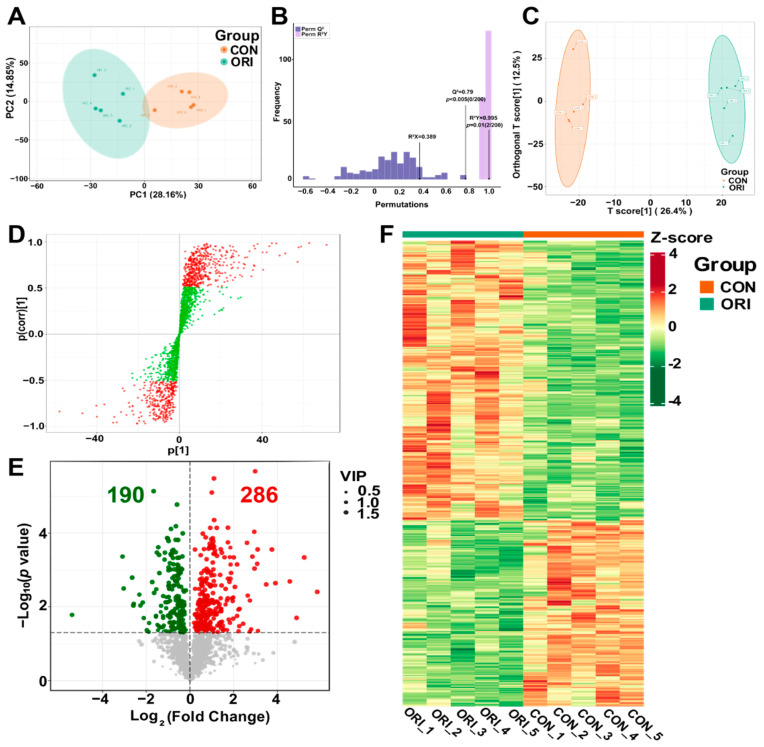
(**A**) PCA plots between the oridonin and control groups. (**B**) The predictive ability of the OPLS-DA model is reliable. (**C**) The OPLS-DA model was able to clearly separate the oridonin group from the control group. (**D**) OPLS-DA S-plot. Red dots indicate metabolites with VIP values greater than 1, while green dots indicate metabolites with VIP values less than or equal to 1. (**E**) Volcano plots showing upregulated and downregulated metabolites. (**F**) Correlation heatmap of DEMs.

Metabolites in the samples were detected using LC-MS/MS, with PCA plots (Figure 7A) showing substantial variances in metabolite compositions between the two groups. Moreover, the OPLS-DA analysis demonstrated effective differentiation of the oridonin group from the control group. Results of the permutation test showed R^2^X = 0.389, R^2^Y = 0.995, and Q^2^ = 0.79, confirming the reliability of the predictive ability of the OPLS-DA model (Figure 7B,C).

The OPLS-DA S-plot displays metabolites with significant differences based on VIP values (Figure 7D). Metabolites positioned closer to the upper right and lower left corners indicate more pronounced variances. In this plot, red dots represent metabolites with VIP > 1, while green dots represent those with VIP ≤ 1. According to the VIP values in the OPLS-DA model variables, where *p* < 0.05 and VIP > 1, 476 differentially expressed metabolites (DEMs) were identified, comprising 190 downregulated and 286 upregulated metabolites (Figure 7E). As shown in the volcano diagram, green represents downregulated changes and red represents upregulated changes. In order to express the clustering relationships between the two groups, 476 DEMs were selected to construct a heatmap (Figure 7F). The information of 476 DEMs is listed in Table S5.

**Figure 8:**
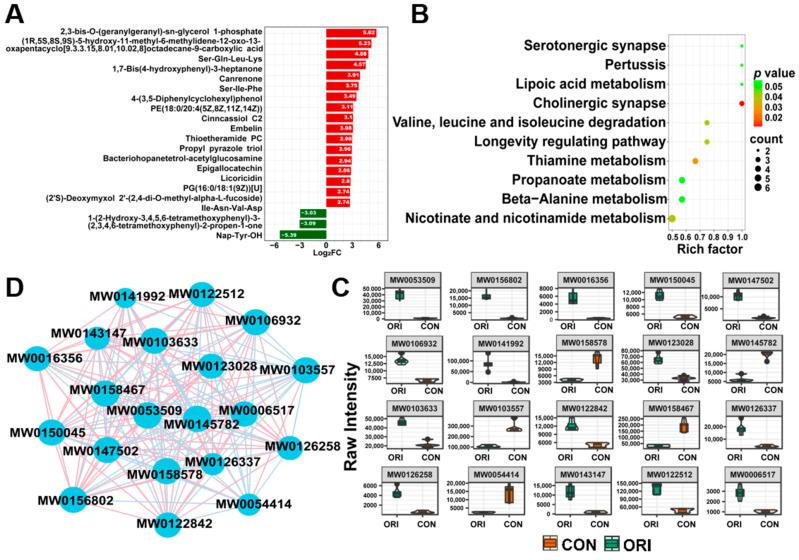
(**A**) Bar graph of DEM multiplicity, presenting the top 20 metabolites with the highest difference in multiplicity. (**B**) The top 10 enriched KEGG pathways of DEMs displayed by *p*-value in the oridonin and control groups (*p* < 0.05, VIP > 1). (**C**) Correlation analysis among samples. The top 20 differential metabolites with the highest VIP values are included. (**D**) Violin plot showing the top 20 DEMs based on VIP values.

Among the differentially accumulated metabolites, 2,3-bis-0-(geranylgeranyl)-sn-glycerol 1-phosphate had the highest |log_2_Fold Change| values (Figure 8A). The analysis of the differential metabolites identified in the previous screening was conducted using KEGG metabolic database; the primary pathways in these differential metabolites were cholinergic synapse, thiamine metabolism, longevity regulating pathways, and valine, leucine, and isoleucine degradation pathways (Figure 8B). Figure 8C illustrates a differential metabolite correlation network diagram, showing the top 20 metabolites with the largest VIP values. In this diagram, pink lines represent positive correlations, while blue lines indicated negative correlations. The thickness of the lines reflects the absolute value of the correlation coefficient; thicker lines denote stronger correlations. A violin plot (Figure 8D) displays the top 20 metabolites with the greatest differential multiplicity, with red indicating upregulated and green indicating downregulated. The names of the 20 metabolites corresponding to their IDs are listed in Table S6.

### 3.9. Oxidative Stress and DNA Damage Induced by Oridonin

**Figure 9:**
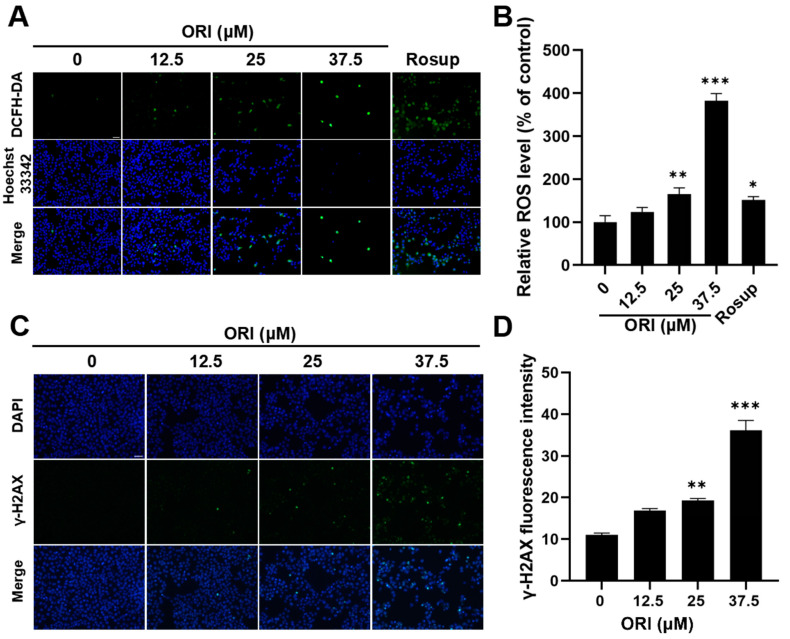
(**A**) Representative images showing the intracellular ROS production. (**B**) Analysis of the relative fluorescence intensity of DCF in the oridonin group and the control group. Immunofluorescence analysis (**C**) and quantitative analysis (**D**) of phosphorylated γ-H2AX induced by 0, 12.5, 25, and 37.5 μM oridonin. * *p* < 0.05, ** *p* < 0.01, and *** *p* < 0.001, n = 3. Scale bar = 100 μm.

Oridonin-induced oxidative damage in HTR-8/SVneo cells was evaluated by measuring the level of intracellular ROS. After a 24 h exposure, the fluorescence intensity of DCF, which indicates the level of ROS production, exhibited a significant rise from 100% in the control group to 123.6%, 165.2%, and 382.7% in the 12.5, 25, and 37.5 μM oridonin groups (Figure 9A,B). The excessive accumulation of intracellular ROS is considered detrimental to the cells.

γ-H2AX acts as a highly sensitive marker for detecting DNA damage that leads to double-strand breaks. Figure 9C,D demonstrate that treatment with oridonin resulted in intensified green fluorescence and a notable increase in the level of γ-H2AX, indicating DNA damage occurred in HTR-8/SVneo cells.

### 3.10. The Adverse Impacts of Oridonin Therapy on Mitochondrial Function

**Figure 10:**
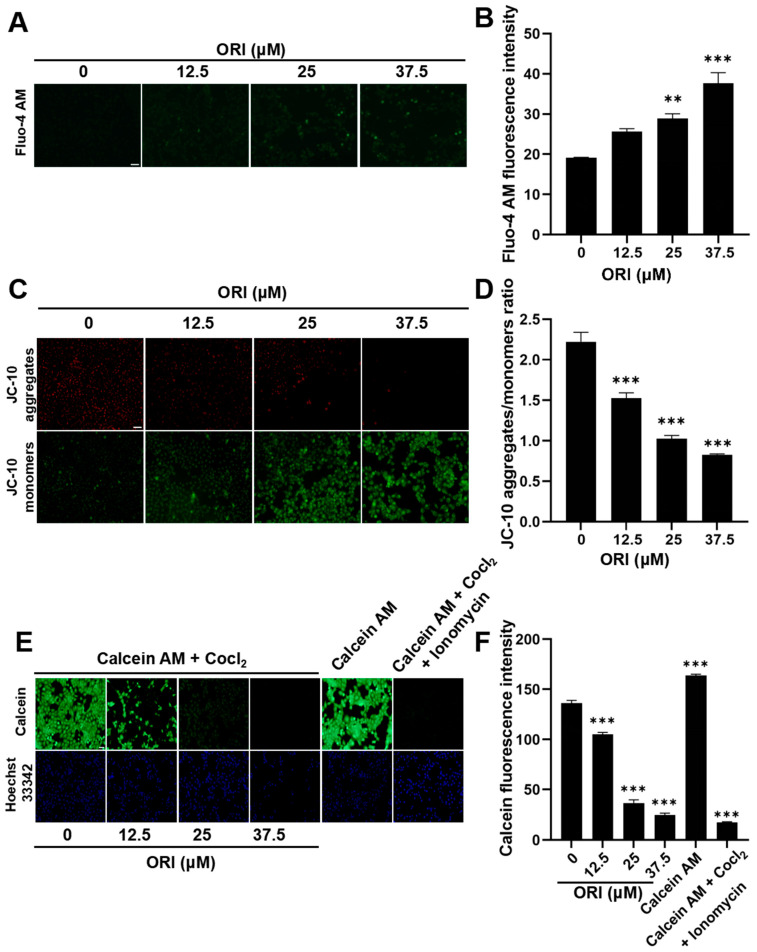
(**A**) Intracellular Ca^2+^ was visualized using Fluo-4 AM in fluorescence microscopy images. (**B**) Statistics of intracellular Ca^2+^ level. Oridonin induced mitochondrial damage in HTR-8/SVneo cells. JC-10 (**C**) and Calcein AM (**E**) probes were used to detect the MMP and mPTP in oridonin-treated HTR-8/SVneo cells, respectively. Quantitative results of the JC-10 probe (**D**) and Calcein AM probe (**F**) in oridonin-treated HTR-8/SVneo cells. ** *p* < 0.01, *** *p* < 0.001, n = 3. Scale bar = 100 μm.

In this study, intracellular Ca^2+^ levels were measured by fluorescence probe Fluo-4 AM. Compared with the control group, exposure of HTR-8/SVneo cells to oridonin increased intracellular Ca^2+^ levels (Figure 10A,B), and revealed the overload of intracellular calcium ions. Figure 10C,D illustrate a decreasing trend in the red–green fluorescence ratio, which was measured in the 12.5, 25, and 37.5 μM groups compared to the control group (*p* <0.05), with values of 1.53 ± 0.07, 1.03 ± 0.04, and 0.83 ± 0.01, respectively. This indicated that oridonin treatment leads to a decrease in MMP in HTR-8/SVneo cells. The fluorescence intensities of Calcein were measured in different groups in Figure 10E,F. The obtained results demonstrated that the fluorescence intensities of Calcein were 136.14 ± 2.85, 105.16 ± 1.85, 36.58 ± 3.14, and 24.73 ± 1.85 in the 0, 12.5, 25, and 37.5 μM oridonin groups, respectively. This result indicated that oridonin treatment led to a dose-dependent reduction in the green fluorescence intensity of Calcein, suggesting a notable induction of mPTP opening.

### 3.11. Expression Levels of mRNA and Proteins

**Figure 11:**
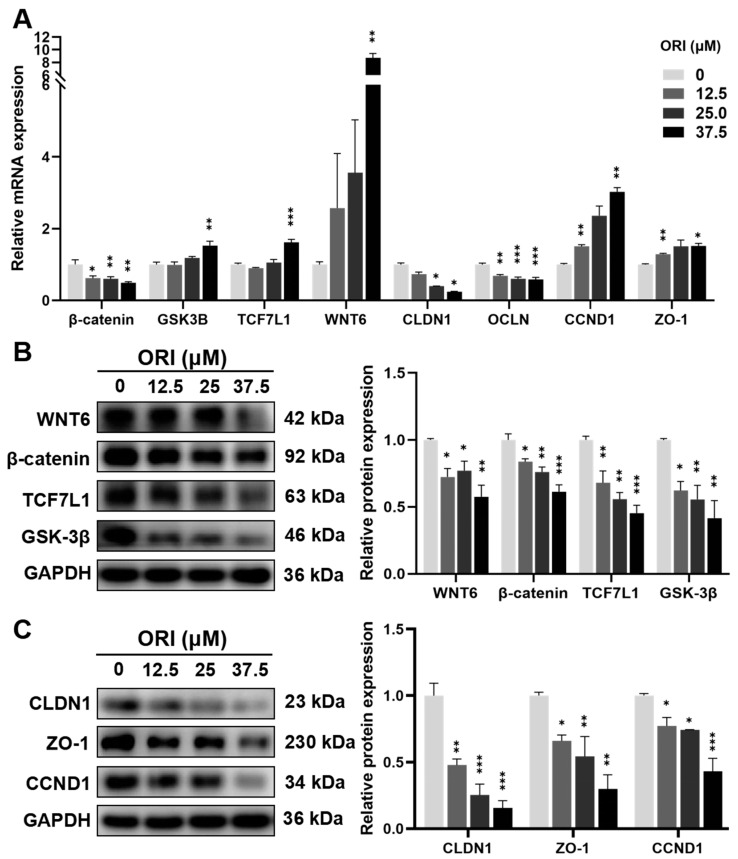
(**A**) The mRNA levels were measured through RT-qPCR analysis. (**B**) Expression levels of WNT6, β-catenin, TCF7L1, and GSK-3β proteins in 0, 12.5, 25, and 37.5 μM oridonin-treated HTR-8/SVneo cells. (**C**) Protein expression levels of CLDIN1, ZO-1, and CCND1 in 0, 12.5, 25, and 37.5 μM oridonin-treated HTR-8/SVneo cells. * *p* < 0.05, ** *p* < 0.01, and *** *p* < 0.001, n = 3.

In order to demonstrate the high correlation of Wnt signaling pathway, cell cycle, and tight junction with the reproductive toxicity of oridonin, we selected pivotal genes and proteins associated with these terms for experimental validation. RT-qPCR was employed to evaluate the mRNA expression levels of these pivotal genes. The results depicted in Figure 11A demonstrate that oridonin led to a decrease in the expression of *β-catenin*, *CLDN1,* and *OCLN* in HTR-8/SVneo cells, while increasing expression of *GSK3B*, *TCF7L1*, *WNT-6*, *CCND1*, and *ZO-1*.

As shown in Figure 11B,C, results of Western blotting indicated a significant reduction in the expression level of WNT6, β-catenin, TCF7L1, and GSK-3β in the oridonin group compared to the control group. Additionally, the levels of CLDN1, ZO-1, and CCND1 also decreased in response to oridonin.

## 4. Discussion

Traditional Chinese medicine has long been recognized for its safety. However, there have been frequent reports of adverse reactions in recent years. Oridonin is a tetracyclic diterpene compound extracted from *Rabdosia rubescens*, which exhibits anti-inflammatory [42] and anti-tumor effects [43]. However, studies have shown that oridonin promotes apoptosis by inhibiting nuclear factor-κB activation and causing G2/M phase arrest [44,45,46,47]. Furthermore, oridonin inhibits caspase-9 and activates TP53-related and PI3K/Akt pathways to promote ROS accumulation, resulting in cell death [48,49]. Therefore, these reports suggest that attention should be paid to the safety problems associated with oridonin in clinical practice. Although the toxic effects of oridonin have been reported, the exact toxicity mechanisms remain incompletely understood.

The combination of multiple omics methods has been utilized to investigate the mechanisms and potential biomarkers of reproductive toxicity induced by Chinese medicine components. In a study on testicular injury induced by *Tripterygium wilfordii* polyglycoside tablet (TWP) in rats, it was observed that TWP significantly reduced the protein levels of ZO-1, OCT4, CLDN11, and PLZF, indicating dysfunction of the blood–testis barrier and impairment of spermatogenesis. Furthermore, levels of ferroptosis-related proteins NRF2, SLC7A11, and GPX4 were notably decreased, while the expression level of 4-HNE was elevated. Additionally, the integrated analysis of DEGs and altered metabolites revealed the crucial roles of ferroptosis and glutathione metabolism in testicular injury [50]. Our study successfully constructed a comprehensive multiomics map of oridonin and identified the signaling pathways and biomarkers. Utilizing epigenetic transcriptome and proteome data, we uncovered key signaling pathways including the Wnt signaling pathway, tight junction, and focal adhesion. m6A regulators confer biological functions to m6A in RNA, precisely regulating balance under stimulation to maintain proper cellular metabolism. Defects in m6A methylation or demethylation may lead to severe physiological consequences, such as abnormal reproductive development in mammals. The study indicated that treatment with oridonin increased the expression of FRM1, a ribosome-associated RNA-binding protein involved in follicular genesis. The expression of FRM1 was notably upregulated in patients with premature ovarian insufficiency [51]. Metabolomics analysis indicated that differential metabolites mainly participated in thiamine metabolism, longevity regulating pathway, and valine, leucine, and isoleucine degradation pathway. Adequate thiamine levels help sustain maternal and fetal mitochondrial function, ensuring normal cell division and growth progression, particularly in germ cell maturation [52].

Oridonin has been found to exert effects across multiple cellular levels [43,53,54]. Numerous investigations have associated oridonin toxicity with the modification of essential biomolecule structure and function through involvement in ROS generation [43,44,55]. Ca^2+^ ions serve as multifunctional second messengers, and participate in various intracellular processes, including signal transduction, muscle contraction, and oxidative stress. Treatment of HTR-8/SVneo cells with oridonin increased Ca^2+^ levels and demonstrated the presence of mitochondrial dysfunction and oxidative stress. Free radicals and oxidative molecules produced during oxidative stress can trigger DNA damage. Exposure of human colorectal cancer cells SW1116 and SW480 to alantolactone caused a substantial increase in ROS level, leading to profound oxidative DNA damage [56]. The results indicated that oridonin triggered HTR-8/SVneo cell ROS accumulation and DNA damage. The study demonstrated that reproductive toxicity was mediated by oridonin, as shown by increased ROS levels, elevated LDH leakage, reduced MMP, and changes in WNT6, β-catenin, TCF7L1, GSK-3β, CLDN1, ZO-1, and CCND1 mRNA and protein expression. We confirmed through RT-qPCR and Western blotting that these factors associated with the Wnt signaling pathway, cell cycle, and tight junctions were highly correlated with the reproductive toxicity of oridonin. However, the observed mRNA and protein expression levels in our experimental results were inconsistent with those obtained from transcriptomics and proteomics analysis, possibly due to differences in technical sensitivity between omics approaches and experimental methods. Oxidative damage leads to mitochondrial dysfunction, which is characterized by alterations in mitochondrial morphology and impaired mitochondrial function. Pathological conditions often give rise to structural changes in mitochondria, including swelling and fragmentation, with increased mitochondrial fission. A significant indicator of mitochondrial dysfunction is the disruption of the MMP. Loss of MMP leads to dysfunction in the mitochondrial electron transport chain, diminished oxygen consumption for metabolism, ATP depletion, and compromised energy metabolism. More specifically, the opening of the mPTP leads to the liberation of cytochrome c from mitochondria into the cytoplasm, thereby activating pro-apoptotic caspases. Mitochondrial dysfunction is frequently associated with disruptions in calcium homeostasis and mutations in mitochondrial DNA [57]. The Wnt/β-catenin signaling pathway serves as a crucial controller of cell proliferation and maintains the pluripotent state in embryonic stem cells (ESCs). Dysregulation of the Wnt signaling pathway during early development stages gives rise to a range of hereditary diseases that result in abnormalities in embryonic development [58]. To further understand the mechanism of reproductive toxicity induced by oridonin, the status of the Wnt signaling pathway was examined. The Wnt/β-catenin signaling pathway is crucial and highly conserved in embryonic development and is also implicated in carcinogenesis due to its involvement in regulating cell differentiation, proliferation, and migration [59]. Berberine antagonized the increase in FITC-dextran permeability in rat intestinal microvascular endothelial cells caused by LPS and upregulated the expression levels of VE-cadherin, β-catenin, and claudin-12 [60]. Furthermore, the upregulation of Wnt1 expression in C57MG mammary tumor-derived cell line results in elevated levels of Connexin43 protein expression [61]. In this study, the downregulation of tight junction-associated proteins CLDN1 and ZO1 led to damage in HTR-8/SVneo cell junctions caused by oridonin. This damage may have resulted in injury to the epithelial barrier, restricting embryo growth and development. According to the transcriptomics, proteomics, and metabolomics analysis, the Wnt/β-catenin signaling pathway is one of the most relevant pathways involved in the adverse reproductive effects of oridonin. In the current study, a notable decrease in the expression of Wnt6 and β-catenin proteins was observed in HTR-8/SVneo cells following treatment with oridonin, as compared to the control group. This discovery aligns with a recent investigation that demonstrated the persistent activation of the Wnt signaling pathway during oridonin-induced reproductive toxicity injury. These results indicate that inhibiting the Wnt/β-catenin signaling pathway and disruption of tight junction are associated with oridonin-induced cytotoxicity and mitochondrial dysfunction.

## 5. Conclusions

The study identified that oridonin can induce reproductive toxicity by eliciting oxidative stress, DNA damage, and mitochondrial dysfunction. The integration of multi-omics approaches revealed the participation of the Wnt/β-catenin signaling pathway and tight junction in the damage caused by oridonin to HTR-8/SVneo cells. The mechanism uncovered in this study may provide new strategies to mitigate oridonin-induced reproductive toxicity.

## Figures and Tables

**Table 1:** The RT-qPCR primer sequences employed in this study.

Gene	Forward Primer (5′→3′)	Reverse Primer (5′→3′)
*GSK3B*	GCACTCTTCAACTTCACCACTCAAG (F)	CTGTCCACGGTCTCCAGTATTAGC (R)
*β-catenin*	ATAGAGGCTCTTGTGCGTACTGTC (F)	TTGGTGTCGGCTGGTCAGATG (R)
*WNT-6*	TGCCAGTTCCAGTTCCGCTTC (F)	CCGTCTCCCGAATGTCCTGTTG (R)
*CLDN1*	AGCCAAGGTGTTGACTCAGACTC (F)	AGCCTCCGCATTAGTTCCATAGC (R)
*TCF7L1*	GGAGCCGAGCAGCGATAGC (F)	CCTCTCCGCCTCCGAGTCC (R)
*ZO-1*	GCGGATGGTGCTACAAGTGATG (F)	GCCTTCTGTGTCTGTGTCTTCATAG (R)
*CCND1*	CGCCCTCGGTGTCCTACTTC (F)	GACCTCCTCCTCGCACTTCTG (R)
*OCLN*	ACTTCGCCTGTGGATGACTTCAG (F)	TTCTCTTTGACCTTCCTGCTCTTCC (R)
*GAPDH*	TGACATCAAGAAGGTGGTGAAGCAG (F)	GTGTCGCTGTTGAAGTCAGAGGAG (R)

**Table 2:** The mRNA expression levels of m6A regulators in oridonin-treated HTR-8/SVneo cells.

Genes	Regulation	Base Mean	log_2_Fold Change	*p* Value
*IGF2BP2*	reader	12,299	−0.42	6.2 × 10^−16^
*FMR1*	reader	5256	0.44	1.2 × 10^−5^
*HNRNPA2B1*	reader	190,433	0.27	1.4 × 10^−4^
*RBM15B*	writer	4641	−0.15	3.2 × 10^−3^
*WTAP*	writer	4354	−0.19	1.2 × 10^−2^
*RBM15*	writer	810	−0.68	2.3 × 10^−2^
*YTHDF2*	reader	1870	0.17	3.2 × 10^−2^
*IGF2BP1*	reader	8764	−0.22	5.6 × 10^−2^
*HNRNPC*	reader	82,291	−0.09	7.4 × 10^−2^
*YTHDF3*	reader	883	1.15	1.1 × 10^−1^
*ZC3H13*	writer	47,093	0.12	1.3 × 10^−1^
*IGF2BP3*	reader	2040	−0.12	1.6 × 10^−1^
*FTO*	eraser	1580	0.14	1.7 × 10^−1^
*ALKBH5*	eraser	16,193	0.08	2.4 × 10^−1^
*YTHDC2*	reader	1778	−0.09	2.4 × 10^−1^
*METTL14*	writer	5153	−0.06	3.5 × 10^−1^
*METTL5*	writer	2978	−0.06	3.7 × 10^−1^
*YTHDF1*	reader	3137	0.04	4.6 × 10^−1^
*CBLL1*	writer	1580	0.03	7.7 × 10^−1^
*YTHDC1*	reader	18,686	−0.08	8.0 × 10^−1^
*VIRMA*	writer	10,151	0.01	8.3 × 10^−1^
*METTL3*	writer	1885	0.01	9.4 × 10^−1^

**Table 3:** The interactions between oridonin and m6A regulatory proteins.

Protein	PDB ID	Total Score	Crash	Polar	H-Bond Number	Residues Involve in H-Bond Formation	Hydrophobic Contact Number	Residues Involve in Hydrophobic Contacts
FMR1	2QND	6.5681	−0.9508	6.7019	5	Arg11, Asp13, Glu68 (3 H-bonds)	2	Arg100, Glu104
HNRNPA2B1	5WWG	5.9849	−4.3987	3.4371	3	Glu101, Lys104, Ala107	8	Pro105, Gly106, Lys186, Arg185, Val170, Lys173, Leu171, Val197
IGF2BP2	6ROL	4.432	−0.8333	3.5298	4	Lys509 (3 H-bonds), Asn503	5	Phe502, Leu510, Glu511, Ile562, Gol604
RBM15	7Z27	4.1057	−0.6563	3.4976	4	Lys9, Lys51, Leu12 (2 H-bonds)	2	Ala13, Gln15
WTAP	7YFJ	3.6498	−0.9403	2.5236	3	Gln61 (3 H-bonds)	3	Tyr64, Leu68, Ser65
YTHDF2	4WQN	3.1273	−0.587	2.3001	2	Arg425, Lys428	2	Asp421, His424

## Data Availability

Data are contained within the article and Supplementary Materials.

## Supplementary Materials

The following supporting information can be downloaded at: https://www.mdpi.com/article/10.3390/toxics12050339/s1, Table S1: Summary of mRNA sequence reads from HTR-8/SVneo cells with or without oridonin treatment. Table S2: 4039 differentially modified m6A genes were obtained after treating with 25 μM oridonin for 24 h in HTR-8/SVneo cells. Table S3: 2996 DEGs were obtained after treating with 25 μM oridonin for 24 h in HTR-8/SVneo cells. Table S4: 62 DEPs were obtained after treating with 25 μM oridonin for 24 h in HTR-8/SVneo cells. Table S5: 476 DEMs were obtained after treating with 25 μM oridonin for 24 h in HTR-8/SVneo cells. Table S6: The names of 20 metabolites corresponding to the IDs.
