# Supplementary material for: Integrating Epigenetics, Proteomics, and Metabolomics to Reveal the Involvement of Wnt/β-Catenin Signaling Pathway in Oridonin-Induced Reproductive Toxicity
Source: Toxics. 2024 May 7;12(5):339. doi: 10.3390/toxics12050339 (PMC11126149; doi:10.3390/toxics12050339)
Supplement: Supplementary file 1 [file toxics-12-00339-s001.zip › toxics-2958185-supplementary/Supplementary File/Table S6.pdf]

Table S6. The names of 20 metabolites corresponding to the IDs

Index	Compounds	Index	Compounds
MW0053509	(1R,5S,8S,9S)-5-hydroxy-11-methyl-6-methylidene-12-oxo-13-oxapentacyclo[9.3.3.15,8.01,10.02,8]octadecane-9-carboxylic acid	MW0103633	Nicotinate mononucleotide
MW0156802	Ser-Ile-Phe	MW0103557	Flavin adenine dinucleotide
MW0016356	Canrenone	MW0122842	Amlodipine
MW0150045	Glu-Leu	MW0158467	Tyr-Glu-Val-Lys
MW0147502	Cinncassiol C2	MW0126337	Pyridoxamine phosphate
MW0106932	Glu-Val	MW0126258	Propyl pyrazole triol
MW0141992	2,3-bis-O-(geranylgeranyl)-sn-glycerol 1-phosphate	MW0054414	L-Palmitoylcarnitine
MW0158578	Tyr-Phe-Thr-Lys	MW0143147	4-(3,5-Diphenylcyclohexyl)phenol
MW0123028	Bendamustine hydrochloride	MW0122512	7-methyl-2-morpholino-9-(1-(phenylamino)ethyl)-4H-pyrido[1,2-a]pyrimidin-4-one
MW0145782	Asn-Ile-Arg-Asp	MW0006517	CAY10448
